# PIM Kinases and Their Relevance to the PI3K/AKT/mTOR Pathway in the Regulation of Ovarian Cancer

**DOI:** 10.3390/biom8010007

**Published:** 2018-02-04

**Authors:** Aziz Ur Rehman Aziz, Sumbal Farid, Kairong Qin, Hanqin Wang, Bo Liu

**Affiliations:** 1 Department of Biomedical Engineering, Dalian University of Technology, Dalian 116024, China; azizjatoi@hotmail.com (A.U.R.A.); krqin@dlut.edu.cn (K.Q.); 2 State Key Laboratory of Fine Chemicals, Dalian University of Technology, Dalian 116024, China; sumbalsrg@mail.dlut.edu.cn; 3 Center for Translational Medicine, Suizhou Hospital, Hubei University of Medicine, Suizhou 441300, China

**Keywords:** PI3K/AKT/mTOR pathway, BAD, cMyc, CDK1

## Abstract

Ovarian cancer is a medical term that includes a number of tumors with different molecular biology, phenotypes, tumor progression, etiology, and even different diagnosis. Some specific treatments are required to address this heterogeneity of ovarian cancer, thus molecular characterization may provide an important tool for this purpose. On a molecular level, proviral-integration site for Moloney-murine leukemia virus (PIM) kinases are over expressed in ovarian cancer and play a vital role in the regulation of different proteins responsible for this tumorigenesis. Likewise, the phosphoinositide 3-kinase (PI3K)/protein kinase B (AKT)/mammalian target of rapamycin (mTOR) pathway is also a central regulator of the ovarian cancer. Interestingly, recent research has linked the PIM kinases to the PI3K/AKT/mTOR pathway in several types of cancers, but their connection in ovarian cancer has not been studied yet. Once the exact relationship of PIM kinases with the PI3K/AKT/mTOR pathway is acquired in ovarian cancer, it will hopefully provide effective treatments on a molecular level. This review mainly focuses on the role of PIM kinases in ovarian cancer and their interactions with proteins involved in its progression. In addition, this review suggests a connection between the PIM kinases and the PI3K/AKT/mTOR pathway and their parallel mechanism in the regulation of ovarian cancer.

## 1. Introduction

Ovarian cancer (OC) is estimated as the fifth most common cause of cancer death in women with a survival-rate of less than 30% [1]. In 2017, the American Cancer Society estimated 22,440 cases diagnosed with OC and 14,080 deaths because of OC in United States [2]. This case-to-fatality ratio makes OC as the most lethal gynecologic-cancer. This high mortality rate by OC is due to less-effective diagnostic tools/screening programs and higher heterogeneity of OC, reviewed in [3]. Most treatment strategies have targeted OC as one disease but OC comprises various types of tumors that differ in their morphology, etiology, molecular biology and prognosis. OC is highly heterogeneous based on its origin in addition to genomic characterization, reviewed in [4]. On the basis of cellular origin, more than 30 types of OC are classified into three groups, including epithelial tumors (85–90%), germ cell carcinoma tumors (5%) and stromal carcinoma tumors (5%) originate from epithelial cells, germ cells and stromal cells, respectively. OC ranges from stage I to stage IV, as a rule, and a higher number indicates the more the cancer has spread [5]. Unfortunately, more than two thirds of OC patients are diagnosed at advanced stages and current standard strategies are not effective enough to treat them. Thus, there is a dire need to develop some novel strategic tools with molecular background to deal with OC [6].

**Figure 1:**
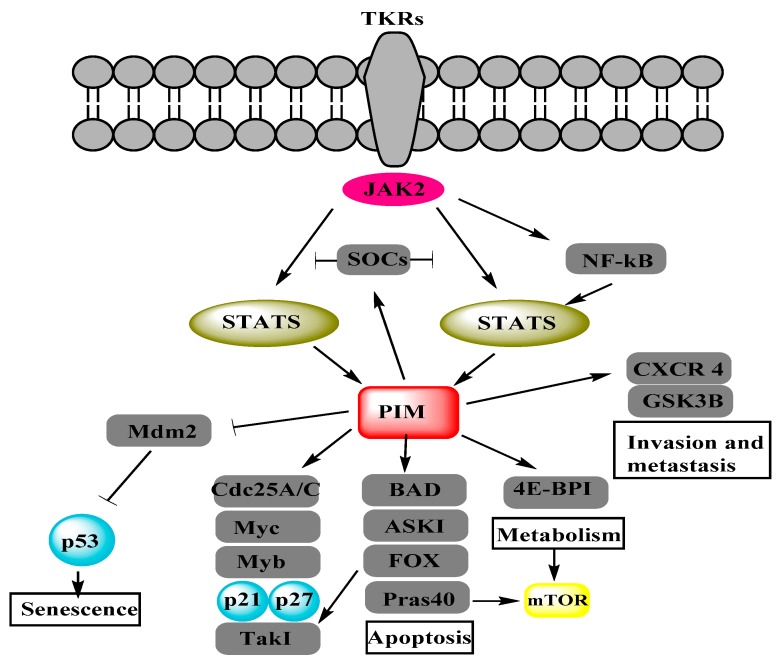
Proviral-integration site for Moloney-murine leukemia virus (PIM) kinases regulate different cancerous pathways by the phosphorylation of target proteins. Adapted from Figure 2 of [7]. Janus kinase (JAK), nuclear factor of kappa light chain gene enhancer in B cells (NF-κB), suppressor of cytokine signaling (SOCS), signal transducer and activator of transcription (STAT), BCL2 antagonist of cell death (BAD), cell division cycle 25 (Cdc25), eukaryotic translation initiation factor 4E-binding protein 1 (4E-BP1), mammalian target of rapamycin (mTOR), apoptosis signal-regulating kinase (ASK1), glycogen synthase kinase-3 beta (GSK3β), avian myelocytomatosis viral oncogene homolog (Myc), forkhead box (FOX), proline-rich AKT substrate (Pras40), transforming growth factor-beta-activated kinase 1 (Tak1), murine double minutes 2 (Mdm2).

Proviral-integration site for Moloney-murine leukemia virus (PIM) proteins i.e., PIM1, PIM2 and PIM3 belong to a family of serine/threonine protein kinases and depend on Ca^2+^/calmodulin protein kinase group. These kinases are short lived and active with highly overlapping functions, reviewed in [7]. They play a vital role in the malignant transformations, subsequent growths, cell cycle regulation, anti-apoptotic activity, metastasis and proliferations of different cancers, reviewed in [8,9]. Figure 1 shows the PIM-regulated pathways/processes [7] in different cancer cells. Similarly, some studies have associated PIM kinases to OC regulation. PIM1 has been found to mediate the biological effects of SGI-1776, ATP competitive inhibitor of PIM1, in human OC which suggests PIM1 as a novel target for OC [10]. PIM3 over expression in OC cells enhances metastasis-associated in colon cancer-1 (MACC1) mRNA and protein expressions, which promote the SKOV3 cell line migration and proliferation. It suggests that PIM3 is a putative oncogene in SKOV3 cell line [11]. SKOV3 cell line has been characterized as “unlikely high grade” and it cannot be a good model to study OC [12]. Therefore, some other high-grade OC cell lines should be studied for more reliable results. Cisplatin affects PIM2 in OC cell lines more than PIM1 and PIM3. PIM2 inhibition impairs the cell growth and reduces the cisplatin-induced phosphorylation of Bcl-2-associated death (BAD) promoter protein. Moreover, PIM2 inhibition also sensitizes the OC cells to drug induced apoptosis [13]. OC cell lines express the smaller isoform of PIM1 (33 kDa), which is involved in paclitaxel resistance [14]. These results present the imperative role of PIM kinases in OC regulation on a molecular level. On the other hand, the phosphoinositide 3-kinase (PI3K)/protein kinase B (AKT)/mammalian target of rapamycin (mTOR) network is considered to be one of the main pathways involved in OC progression, reviewed in [6,15,16]. PIM kinases have been found to sustain activity of the PI3K/AKT/mTOR pathway [17]. They can cause alterations in the PI3K/AKT/mTOR pathway by influencing PI3K via insulin receptor substrates (IRS) [18] and its downstream component AKT through ROS [19]. PIM and AKT control cell growth and translation through overlapping mechanisms and phosphorylate multiple common substrates to control mTORC1 [8]. These components of the PI3K/AKT/mTOR pathway also play a crucial role in OC [20]. The interactions of PIM kinases with each component of the PI3K/AKT/mTOR pathway propose that PIM kinases may regulate OC either directly or/and through the PI3K/AKT/mTOR pathway.

In this review, role of PIM kinases in the regulation of OC and their biological effects on different proteins involved in OC are discussed. In addition, an overview of the PI3K/AKT/mTOR network and interactions of its downstream components with PIM kinases in different cancer cells is presented, specially focusing the OC cells. This will provide better understanding of the mechanistic approach of OC regulation by PIM kinases and the PI3K/AKT/mTOR pathway. Hopefully, this knowledge may guide researchers to develop or/and improve treatment strategies dealing with drug resistance, predictive markers, diagnosis, and treatment of OC, particularly at a molecular level.

## 2. Interrelation of PI3K/AKT/mTOR and PIM Pathways in Other Cancers

The PI3K/AKT/mTOR signaling pathway is a central regulator of both normal and cancerous cell physiology that is altered in many human cancers [21,22,23,24]. It controls cell cycle, metabolism, cell survival, motility, chemoresistance, angiogenesis, and genomic instability [6]. Interaction of this pathway has been demonstrated with PIM kinases in different cancers, for example, efficiency of triple inhibition of PIM, PI3K and mTOR in mantle cell lymphoma [25] and multiple myeloma [26]. The epidermal growth factor receptor (EGFR) aberrations over activate the PI3K/AKT/mTOR pathway [27] and its irradiation induced activation also upregulates PIM1 in head and neck carcinoma [28]. It mentions that they have same upstream regulator in head and neck carcinoma. Recently, ELF4B has been reported as a common substrate of PIM kinases and the PI3K/AKT/mTOR signaling pathway [29]. Inhibition/downregulation of PIM kinases decreases the phosphorylation of downstream components of the PI3K/AKT/mTOR pathway in glioblastoma [30]. Above data suggests the interrelation of PIM kinases with The PI3K/AKT/mTOR pathway in different cancerous cells. For better understanding, each component of this pathway is discussed below in detail.

### 2.1. PI3K

**Figure 2:**
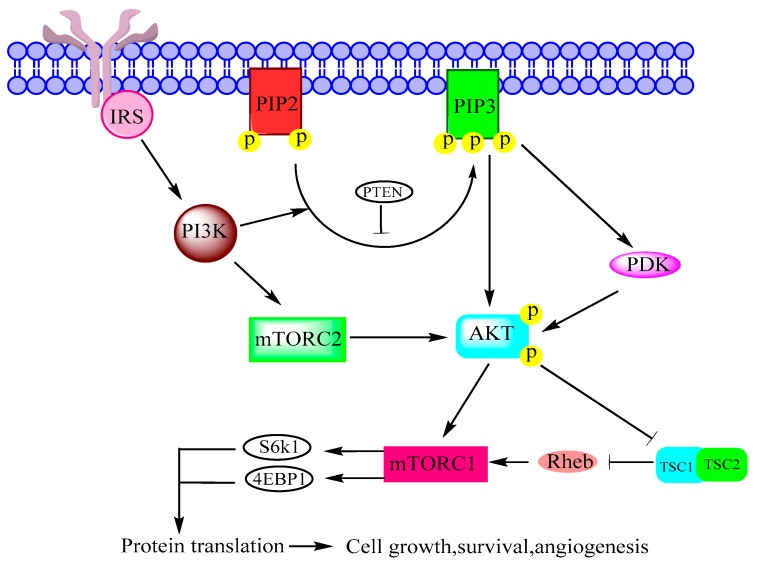
Phosphoinositide 3-kinase (PI3K)/protein kinase B (AKT)/mTOR pathway adapted from figure 2 of [6] and figure 1 of [16]. Insulin receptor substrate (IRS), phosphatase and tensin homolog (PTEN), phosphatidylinositol bisphosphate (PIP2), phosphatidylinositol trisphosphate (PIP3), tuberous sclerosis (TSC), Ras homolog enriched in brain (Rheb), S6 kinase beta-1 (S6K1).

Class I of PI3K is associated with cell proliferation, immune functions, insulin signaling and inflammation [6], and it is frequently mutated in human cancers [31]. Class II regulates membrane trafficking and Class III has important role in autophagy [15]. PI3K converts PIP-2 to PIP-3, which allows AKT and PDK1 to come together near cell membrane. Consequently, AKT is phosphorylated at Thr-308 and at Ser-473 by PDK1 and mTORC2, respectively. AKT directly activates mTORC1 or inhibits TSC1/2 complex for the activation of mTORC1 as shown in Figure 2 [6,16].

PI3K and PIM kinases inhibitors have been used for therapeutic target of different cancers. For example, PIM inhibitor and inhibitor of PI3K catalytic isoform, P110α, together enhance the antineoplastic effects in glioblastoma cells. Similarly, combination of PIM kinases inhibitor ETP-45299 with PI3K inhibitor GDC-0941 is highly synergistic in cells of MV-4-11-AML [32]. It suggests the combined effects of PIM and PI3K in heterogeneous tumors [30].

### 2.2. AKT

AKT, also known as protein kinase B, is a serine/threonine kinase protein. It plays core function in the mechanism of many types of cancers [33,34]. PIM kinases and AKT have the similar regulatory pathway and also control the similar substrates. JAK/STAT provides an alternative signaling system that is mandatory for AKT activation, and this pathway also regulates PIM genes. Consensus phosphorylation motif of AKT and PIM kinases is quite similar and they can also recognize similar substrates. This suggests that they control the similar overlapping signaling pathways [35]. Overlapping activities of both kinases are proved from different studies as inhibitors of both kinases provoke cell cycle arrest in many tumors, reviewed in [8]. Protein synthesis modulation via TSC2 and eIF4B also mentions the parallel regulation of pathways of both kinases [36]. Moreover, AKT-1 activation promotes survival of PIM deficient cells [37]. This suggests that there is a functional link between both kinases. AKT overexpression increases PIM1 in neonatal rat cardiomyocytes [33]. Similarly, AKT is an upstream of PIM1 in endothelial cells but it does not affect PIM2 and PIM3 [34]. In v-Abl transformants, reciprocal signaling between PIM and AKT1 is mentioned by Guo et al. [37]. AZD1208 is a pan inhibitor of PIM kinases and its treatment inhibits the PIM kinase activity and, thereby elevates the ROS levels, which subsequently results in the activation of p38 and AKT [19]. Therefore, inhibition of PIM kinases results in the activation of AKT via ROS. These data mention an indirect mutual interaction between both of these kinases and demand further research to probe actual relationship between these two important kinases. PIM kinases are thought to be the critical mediators of the receptor-tyrosine kinases upregulation, which is induced by inhibition of AKT. Co-targeting of both kinases is proposed in prostate cancer treatment [38]. These all above results describe a synergic, parallel and overlapping effect of AKT and PIM kinases in different cancerous pathways. 

### 2.3. mTOR

**Figure 3:**
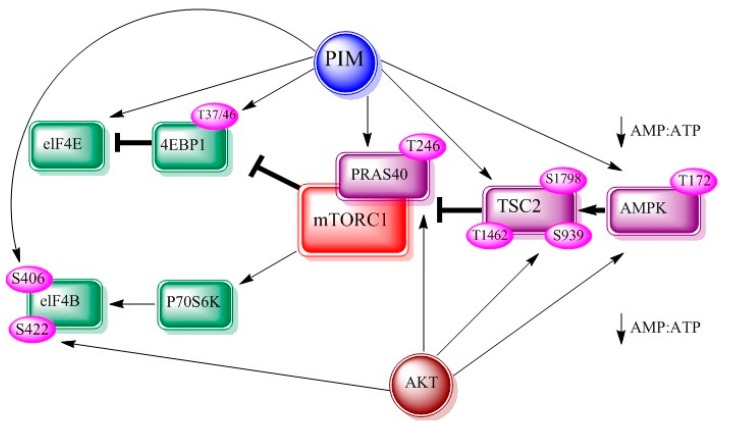
Interrelationship between PIM, AKT and mTOR adapted from figure 2 of [8]. AMP-dependent kinase (AMPK), eukaryotic translation initiation factor 4E (eIF-4E), eukaryotic translation initiation factor 4B (eIF-4B).

mTOR belongs to phosphatidylinositol 3-kinase related kinase family. It is well demonstrated that mTOR is involved in cellular translational machinery, cell growth and cellular metabolism, reviewed in [39,40]. It links other proteins and serves as a catalytic component of the mTORC1 and mTORC2 [41]. PIM and AKT kinases jointly regulate mTOR signaling axis via its effectors. PIM and AKT can activate TSC2 directly [42] or by controlling AMP:ATP ratio [43]. Both kinases phosphorylate proline rich-AKT substrate 40 (PRAS40), which increases mTOR kinase activity on dissociation from mTORC1. Consequently, p70S6 and 4EBP1 kinase phosphorylations are also increased (Figure 3).

PIM kinases expression supports survival of chronic lymphocytic leukemia cells and promotes the CXCR4-mTOR dependent migration [44]. Similarly, mTOR pathway modulation and AZD1208 cytostatic effects are also identified by protein profiling [45]. mTOR regulation by PIM kinases and their interrelation in other cancers advocate their magnitude in tumorigenesis. 

## 3. Interrelation of PI3K/AKT/mTOR and PIM Pathways in Ovarian Cancer

The PI3K/AKT/mTOR pathway is extremely multipart owing to the alterations within this pathway itself and alterations in inputs of the pathway. Due to these changes, this pathway plays a major role in OC tumorigenesis and progression, reviewed in [6,15,16]. A short description of the role of each component of this pathway in OC, and its possible interrelation with PIM kinases is shortly presented below.

### 3.1. PI3K

Active mutations in PI3KCA and PI3KR1 genes, which encode P110α and P85α subunits of PI3K respectively, have been found in OC cells [46,47]. Moreover, 35% of clear cells OC and 20% of endometrioid OC have been reported because of PI3KCA mutations and PTEN loss, respectively [48]. PTEN loss and mutations in PIK3CA have also been applied for OC stimulation in mice [49]. A pan-class I PI3K inhibitors [50], isoform-selective PI3K inhibitors [51] and dual PI3K/mTOR inhibitors are being widely used as a therapeutic target for OC [6]. These results mention the crucial role of PI3K in regulation of OC. Similarly, insulin receptors are also important for the development, survival, maintenance, and chemotherapeutic response of OC. In many studies anti-insulin receptors targeted strategies have reduced the OC models [52]. IRS-1 is the substrate of PIM kinases and it is phosphorylated by these kinases at serine-1101 residue [18]. It is interesting that IRS-1 has been found to affect PI3K [16]. This indicates an indirect relationship between PIM kinases and PI3K through IRS-1. Further research will clarify if IRS-1 is the only linking point between PI3K and PIM kinases or there are some other substances that form a bridge between these two important kinases. 

### 3.2. AKT

AKT plays core function in the mechanisms of many types of cancers [53,54], and its role in OC has also been well described. Activation of the PI3K/AKT pathway stimulates the OC cells proliferation [55] and induces the follicle stimulating hormone driven OC cell proliferation [56]. Moreover, it also mediates OC cells migration and invasion [57], and inhibits the apoptosis and autophagy of OC cells [58,59]. AKT inhibition sensitizes chemoresistant OC cells to cisplatin via abrogating S, and G2/M phase arrest [60]. Furthermore, the P13K/AKT signaling pathway inactivation by Stevioside, a phytochemical, proves the cell cycle arrest in G2/M stage [61]. Role of three isoforms of AKT in OC proliferation, metastasis and angiogenesis are different from each other. AKT-1 is demonstrated as responsible for OC cell proliferation, cell viability and protection from apoptosis [62]. These reports indicate that AKT is a major contributing protein kinase in the progression of OC including cell proliferation, cell growth, migration, invasion, apoptosis and autophagy of OC cells.

As mentioned above that PIM kinases and AKT show parallel and overlapping effects in various cancer cells. Similarly, both kinases show parallel behavior towards the cisplastin in OC cells. PIM kinases [13] and AKT [60] are important in controlling the chemosensitivity of OC cells. PIM can enhance BAD phosphorylation [13] similar to AKT [63,64] in OC cells. It has been reported that PIM kinases can phosphorylate multiple sites on BAD in vitro and in vivo [65]. PIM1 phosphorylates serine-112 at higher rate, while serine-136 and serine-155 at lower rate [65]. PIM2 predominantly phosphorylates serine-112 [65,66]. PIM3 phosphorylates serine-136 and serine-155 more specifically as compared to serine-112. PIM3 can also phosphorylate serine-170 in vitro [65]. While, AKT phosphorylates BAD at serine-136 [63,64]. Besides, inhibition of P21 and P27 proteins by both of the kinases has also been reported in OC cells [10]. Such a parallel behavior of these kinases, in OC cells interrelate the PIM kinases with AKT. However, further studies are required for exploring the synergic and parallel mechanism of these kinases and solving the ambiguities such as PIM kinases either being upstream/downstream of AKT or working through different pathways in OC cells. 

### 3.3. mTOR

mTOR over-activation can cause OC and it is also related to ovarian diseases such as polycystic ovarian syndrome [67]. The mTOR is caught up in OC metastasis and its inhibition causes suppression of OC metastasis [68]. CCL18, a chemotactic cytokine, causes cancer and thought to be a biomarker for epithelial OC. CCL18 induced OC invasiveness is strongly connected with mTORC2 [69]. Blockage of mTOR signaling results in suppression of proliferation of OC [70]. mTOR increases OC cells viability and decreases their apoptosis and autophagy [71]. It is also linked with cisplatin resistance in OC cells [72]. Hence, mTOR is an important regulator of OC. It has also been regulated by PIM kinases in other cancers but the exact mechanism of mTOR and PIM kinases interactions in OC is still debatable.

## 4. PIM Kinases and Proteins Involved in Cellular Functions of Ovarian Cancer Cells

To understand the role of PIM kinases in cellular functions of OC cells deeply, their interactions with some imperative proteins involved in the cellular functions of OC are described in this part. Until now these four important interacting partners of PIM kinases i.e., cMyc, BAD, CDKs and MACC1 have been studied in the OC cells. Roles of these proteins in the OC regulation and their interactions with PIM kinases and the PI3K/AKT/mTOR pathway are reviewed separately in this part. 

### 4.1. cMyc

cMyc initiates and maintains tumorigenesis via the immune regulatory molecules modulation [73], and its discordant expression is found in many tumors [74]. cMyc drives OC cell proliferation, survival and oncogenic potential and its inhibition can disrupt OC malignancy [75]. Significance of cMyc in OC regulation can be evaluated from the fact that higher cMyc mRNA and protein levels have been reported in OC tissues than normal samples. Furthermore, OC cells growth can be inhibited by targeting cMyc [76], and cMyc is also a target for cisplatin resistance in OC [77]. From this data, it can be concluded that cMyc is a vital oncogenic transcription factor for OC pathogenesis. PIM kinases can enhance the ability of cMyc as their inhibition reduces cMyc activity in prostate cancers [78] and multiple myeloma cells [79]. Similarly, the PI3K pathway independent of AKT has also been demonstrated to promote different cancers via mTOR, PDK1 and cMyc [53]. PDK1 independent of PI3K can also cause cMyc phosphorylation in cancer cells [80]. cMyc is considered to be a downstream molecule of the AKT/mTOR/MEK/ERK pathway in renal carcinoma cells [81]. These data suggest the role of PIM kinases and the PI3K pathway and its components in the regulation of cMyc in different cancerous pathways. 

While studying the role of PIM2 on cMyc in OC cells, a pan-PIM inhibitor, SGI-1776, has been used. Different OC cell lines were treated for 48 h with different concentrations of SGI-1776 (0–5 μM). As the inhibition of PIM2 increases, expression of cMyc protein reduces in the OC cells [13]. On the other hand, recently, knocking down of cMyc caused reduction of the PI3K/AKT signaling pathway in OC cells [82] which is opposite to as reported in normal cells [83]. This data suggests the involvement of PI3K, PIM kinases and cMyc together in OC cells but further studies to probe mechanistic approach will help to understand exact relationship between them. 

### 4.2. BAD 

BAD is a pro-apoptotic protein and its phosphorylation is thought to be a critical determinant of OC platinum sensitivity [84] and the BAD apoptosis pathway influences OC chemosensitivity and overall survival [85]. OC cells are resistance to cisplatin (CDDP) treatment and CDDP treatment upregulates the PIM2. Anchorage-independent growth is an important feature of malignant transformations and is a critical factor in the dissemination and metastasis of OC [86]. PIM2 triggers the anchorage-independent growth [13], which results in BAD phosphorylation on serine-75, serine-118 and serine-99 [13]. SGI-1776, which is an inhibitor of all PIM kinases, inhibits the OC cells proliferation with associated reduction in PIM1 kinase activity [10]. Different concentrations of SGI1-776 (2.5, 5, and 10 μmol/L) were used and PIM kinase activity decreased in a dose dependent manner [10]. Pretreatment of OC cells with SGI-1776 (3 μM) also impairs CDDP-induced BAD phosphorylation. Similarly, silencing of PIM2 kinase results impaired BAD phosphorylation with CDDP [13]. Interestingly, BAD phosphorylation is also associated with the PI3K/AKT/mTOR network. AKT can also perform BAD phosphorylation at serine-136 in OC cells [63,64]. These data indicate an interconnection between the AKT pathway and PIM kinases in phosphorylation of BAD. Further research can explore the relevance between action mechanisms of both kinases.

### 4.3. Cyclin-Dependent Kinases 

Cyclin-dependent kinase (CDK) 2 plays an important role in cell-cycle regulation (G1 to S phase transition) and is found involved in cancer progression and proliferation [87,88]. In OC cells, an inverse relation of CDK2 and DNA methylation is observed [89]. CDK2 is thought to be a therapeutic target, but upregulation of its protein causes resistance to its inhibitor in OC cells [90]. CDK6 is associated with platinum sensitivity control through the regulation of FOXO3a-ATR and OC cells proliferation [91]. In addition, cell cycle progression is also suppressed by targeting it [92]. Since CDK4 is also linked with OC cell proliferation and cell-cycle progression, then CDK4/6 inhibitors can be used for OC therapies [93]. This data suggests CDKs are key regulating proteins in OC progression and proliferation. Recently, 1.5–20 μmol/L SGI-1776 was used for 24 h to test its effect on proliferation of OC cells, and decrease in proliferation of OC cells with the inhibition of PIM1 activity was reported [10]. Besides, PIM expression is downregulated with the decrease of CDK6/4/2 and pCDK6/4/2. While CDKs inhibitors (P21 and P27) are increased which finally results in the inhibition of migration, proliferation, and invasion in OC cells [10]. Decrease in proliferation is linked with an increased expression of the CDK inhibitors (P27 and/or P21). This inhibition of PIM1 decreases the cell viability. While, upregulation of the PIM1 with cDNA transfection rescues the inhibition of viability, migration, proliferation of OC cells and their cell-cycle phase 1 accumulation [10]. This data provides the mechanistic evidence that SGI-1776-induces downregulation of PIM1, which inhibits the cell proliferation, invasion and migration of OC cells. Therefore, PIM kinases inactivation would be a good treatment for OC cells progression. Interestingly, inhibition of P21 and P27 by the PI3K/AKT/mTOR/p70S6K1 network in OC cells has also been demonstrated [94], which strongly supports the idea of parallel mechanism of PIM and the PI3K/AKT signaling network in regulation of OC. Further studies are required to find the mechanism of regulation of P21 and P27 by PIM kinases and explore whether it is independent of the PI3K/AKT/mTOR pathway or not.

### 4.4. Metastasis-Associated in Colon Cancer-1

Impaired expression of MACC1 is associated with several malignant tumors. MACC1 down-regulation in OC cells inhibits migration, invasion and proliferation of these cells, and increases the apoptosis [11]. MACC1 inhibition suppresses the growth and metastatic OC cells in vivo and in vitro. In cisplatin resistance epithelial OC cells, MACC1 knockdown increases cisplatin sensitivity [95]. MACC1 can promote metastasis and invasion of OC [96], which is thought to be a predictor of prognosis. Moreover, it is an important therapeutic target of OC [97]. Recently, it has been reported that MACC1 and PIM kinases are correlated to each other and they have similar role. PIM3 and MACC1 [11] expressions have also been reported higher in OC tissues as compared to other tissues [11]. However, the mechanism by which PIM3 regulates the MACC1 expression is still unknown. MACC1 over expression can activate the PI3K/AKT/mTOR pathway in gastric cancer [98]. Interestingly, MACC1 binds to proximal fragment of endogenous MET promoter, and following HGF/MET signaling consequences [99]. On activation, MET can activate PI3K/AKT pathways in OC cells [100]. This data suggests an interconnection between PIM kinases and the PI3K/AKT pathway. 

Above data clearly mentions the role of PIM kinases in the regulation of OC. Expressions of PIM Kinases in OC cell lines [14] suggest their role in the OC cell proliferation, survival and cisplatin resistance via cMyc reduction and BAD phosphorylation [13]. Furthermore, inhibition of OC cells migration, proliferation, invasion and cell-cycle phase 1 accumulation can also be rescued by PIM kinases through CDKs and its inhibitors [10]. MACC1 correlation with PIM kinases and their higher expressions in OC tissues suggest their important role in OC regulation [11]. Similarly, the PI3K/AKT/mTOR network is also linked with these proteins in OC cells, which suggests its connection with PIM kinases. However, these results suggest opening a new door to the mechanistic knowledge of OC and advance studies will be helpful to understand their role properly.

## 5. Summary

**Figure 4:**
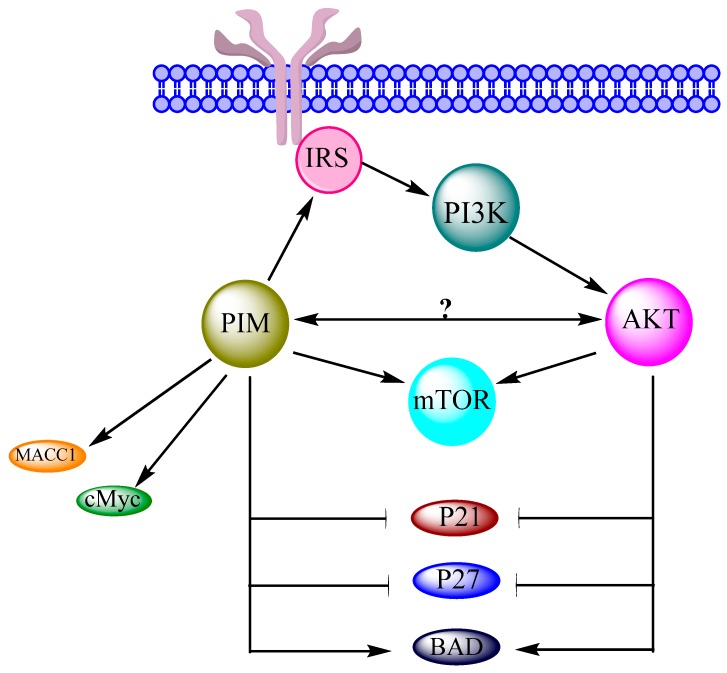
Possible interaction between PI3K/AKT/mTOR network and PIM in ovarian cancer [9,16]. Metastasis-associated in colon cancer-1 (MACC1).

PIM kinases are over expressed in many types of cancers, and different studies also suggest their contribution in the regulation of OC. For understanding the exact mechanism of OC regulation by PIM kinases, a brief knowledge of their interactions with the PI3K/AKT/mTOR network is mandatory because it is the main pathway involved in OC [16]. Indeed, PIM kinases are associated with the components of this cascade in many tumors. Moreover, the JAK/STAT pathway is mandatory for the regulation of both AKT and PIM genes. Both these kinases have similar phosphorylation motif with similar substrates. They are also interconnected in same overlapping signaling pathways [35]. PIM kinases are directly and indirectly found to activate mTOR and its upstream effectors like AKT [8]. A reciprocal signaling mechanism has also been suggested in between PIM and AKT1 in v-Abl transformants [37]. PIM kinases cause phosphorylation of IRS [18], which can alter PI3K. This indicates the close interaction of PIM kinases with PI3K/AKT/mTOR cascade and its components [19]. Similar to cancer cells, PIM kinases also enhance the ability of cMyc in OC cells, which may cause cell proliferation, cell survival and oncogenic potential [13]. Association of the PI3K/AKT/mTOR network with cMyc has also been recently approved in OC. Another important protein, BAD, is phosphorylated by PIM in OC cells. Similarly, AKT has also been reported to perform the BAD phosphorylation in OC cells. PIM and the PI3K/AKT/mTOR network both can inhibit the P21 and P27 expressions in OC cells. MAAC1 expression and PIM expression are found higher in OC cells. Similarly, the PI3K/AKT/mTOR network is activated by over expression of MACC1 in OC. These data suggest a strong possibility of interaction and relevance of PIM kinases and the PI3K/AKT/mTOR network in the regulation of OC (Figure 4).

## 6. Conclusions

Many studies have confirmed the presence of all three of the PIM kinases in OC cells performing different cellular functions. PIM kinase interaction with the PI3K/AKT/mTOR signaling network is confirmed, and their downstream components have shown a close connection with each other in several cancer cells. PIM kinases and the PI3K/AKT/mTOR pathway have also been found to regulate the same proteins involved in OC regulation in the same manner. Similarly, PIM kinases can also affect each component of this pathway directly or indirectly. Such a type of parallel behavior and close connection suggests that PIM kinases may regulate OC either directly or/and through the PI3K/AKT/mTOR pathway. Thus, PIM kinases could be among the key clinical targets to treat OC on molecular levels.
